# Grid partitioning image analysis of highly aggregative bacterium *Acinetobacter* sp. Tol 5

**DOI:** 10.3389/fmicb.2025.1637462

**Published:** 2025-09-10

**Authors:** Yuki Ohara, Shogo Yoshimoto, Katsutoshi Hori

**Affiliations:** ^1^Friend Microbe Inc., Nagoya, Japan; ^2^Department of Biomolecular Engineering, Graduate School of Engineering, Nagoya University, Nagoya, Japan

**Keywords:** microscopy, image analysis, trimeric autotransporter adhesin, cell aggregation, *Acinetobacter*

## Abstract

Bacterial cell aggregation plays a fundamental role in surface colonization, stress tolerance, and interspecies metabolite exchange. Aggregation is assessed by simple tube-settling assays and also image analysis; however, approaches for quantitatively assessing the heterotypic and homotypic cell–cell interactions among more than two types of cells have been limited. In this study, we developed grid partitioning image analysis (GPIA), a simple workflow that quantifies the compositional heterogeneity of bacterial aggregates. Confocal laser scanning microscopy (CLSM) images of fluorescently labeled *Acinetobacter* sp. Tol 5, which exhibits a self-aggregative nature through its cell surface protein AtaA, were partitioned into 2-μm square grids. Grids containing one or no cells were classified as dispersed, whereas those containing multiple cells were classified as aggregates, and the proportion of EGFP-labeled cells within each grid was recorded. Reference images representing dispersed cells, homo-aggregates, and hetero-aggregates produced characteristic EGFP-ratio histograms that matched binomial predictions. When AtaA production in one cell type was decreased, the histogram changed from a symmetric unimodal histogram with the peak at 40–60% EGFP-ratio to a skewed distribution, indicating that GPIA can detect differences in cell-to-cell affinity. Using the same procedure, we examined six in-frame deletion variants of AtaA. The deletion of the N-terminal head domain alone prevented co-aggregation with full-length AtaA, suggesting that homophilic recognition by this domain mediated self-aggregation, whereas deletions in all other regions had no measurable effect. GPIA, therefore, offers a simple and rapid approach for quantitative studies on bacterial cell aggregation, bridging the gap between qualitative microscopy and quantitative but technically demanding single-cell analysis. GPIA will accelerate research on cell–cell interactions, which are the foundational processes that drive biofilm formation and the assembly of microbial consortia.

## 1 Introduction

Bacterial cell aggregation, which includes homotypic aggregation of identical cells and heterotypic aggregation among different types of cells, plays a fundamental role in microbial ecology and pathogenesis (Nwoko and Okeke, 2021; Kragh et al., 2023). By facilitating close cell-to-cell contacts, aggregation promotes initial surface colonization and biofilm formation, shields communities from shear stress, desiccation, and antimicrobials, and accelerates interspecies signaling and metabolite exchange (Kruse et al., 2021; Liu et al., 2024). These cell–cell interactions are mediated by cell-surface adhesins, such as extracellular polysaccharides and protein fibers (Formosa-Dague et al., 2016; Trunk et al., 2018). In the medical field, bacterial cell aggregation is treated as a nuisance (Nwoko and Okeke, 2021; Liu et al., 2024). On the other hand, it is recognized as beneficial in wastewater treatment and bioprocesses for chemical production to concentrate biomass and stabilize the systems (Sethi et al., 2023; Najim et al., 2024; Hammond et al., 2025). Therefore, understanding the characteristics and mechanisms of bacterial cell aggregation is important in various research fields.

*Acinetobacter* sp. Tol 5 shows remarkable nonspecific adhesiveness to various material surfaces and a self-aggregative nature through its cell surface nanofiber protein AtaA (*Acinetobacter* trimeric autotransporter adhesin) (Ishikawa et al., 2012). AtaA is a member of trimeric autotransporter adhesins (TAAs), which are outer membrane proteins widely distributed in gram-negative bacteria (Leo et al., 2012; Meuskens et al., 2019). The polypeptide chains of TAAs form a homo-trimeric structure with an N-terminal passenger domain corresponding to its adhesive functions and a C-terminal transmembrane domain that transports and anchors the passenger domain onto the outer membrane (Lyskowski et al., 2011; Bassler et al., 2015). *Acinetobacter baumannii*, a pathogenic species that has attracted attention as a multidrug-resistant bacterium, also has a TAA called Ata (*Acinetobacter* trimeric autotransporter) (Bentancor et al., 2012; Li et al., 2025). Ata from *A. baumannii* is involved in adhesion to the extracellular matrix, invasion to host cells, and protection against the host (Bentancor et al., 2012; Weidensdorfer et al., 2019; Tram et al., 2021), but there are no reports of Ata being involved in self-aggregation, indicating that there is diversity in the functions of TAAs even within the genus *Acinetobacter*. The adhesive and aggregation features of AtaA can be conferred to other non-adhesive gram-negative bacteria by transformation with the *ataA* gene (Ishikawa et al., 2012; Yoshimoto et al., 2023). Previously, we invented a new method for immobilizing bacterial cells utilizing AtaA (Ishikawa et al., 2014). Large numbers of bacterial cells expressing AtaA can be quickly immobilized onto various material supports and the immobilized cells can be efficiently used for bioproduction (Ishikawa et al., 2014; Yoshimoto et al., 2023). AtaA-mediated self-aggregation plays an important role in the initial attachment of bacterial cells to material surfaces and in increasing the number of immobilized cells by stacking and flocculation (Furuichi et al., 2020). Nevertheless, the details of aggregation remain unclear because bacterial cell aggregation is commonly assessed by simple qualitative tube-settling assays that monitor turbidity (Trunk et al., 2018; Nwoko and Okeke, 2021; Rooke et al., 2021).

Microscopic image analysis is a powerful tool for understanding various characteristics of bacteria and their cell aggregates, including biofilms (Costa et al., 2013; Jeckel and Drescher, 2021). Optical microscopy enables the observation of features such as morphology, size, motility, and other phenotypic traits of individual bacteria and their aggregate structures. Fluorescence *in situ* hybridization (FISH) and fluorescent protein-based reporter systems allow for the spatial visualization and quantification of the proportion and distribution of specific bacterial species (Prudent and Raoult, 2019; Shields et al., 2019; Barbosa et al., 2023). Recently, more quantitative approaches have been developed, including single-cell segmentation, analysis of temporal fluorescence dynamics, and in situ cytometry within biofilms (Paula et al., 2020; Gómez-de-Mariscal et al., 2021; Hartmann et al., 2021). On the other hand, approaches for quantitatively assessing the heterotypic and homotypic cell–cell interactions among more than two species have been limited. Some studies computed, for each cell, the frequency of neighboring cell types to estimate aggregation-pair proportions in mixed-species aggregates (Glass and Riedel-Kruse, 2018; Khalil et al., 2020), but these analyses were complicated and computationally intensive. In contrast, interactions among eukaryotic cells have been assessed with simpler image analyses that calculate the composition of four-cell clusters and sum these values to identify the predominant interaction mode (Sieber and Roseman, 1981). Transferring such simple quantitative imaging strategies to bacteria is beneficial for rapid and intuitive analysis; however, it requires extensive modifications and optimization because bacterial cells are much smaller than eukaryotic cells and form densely packed clusters that obscure individual cell boundaries and confound automated segmentation.

In this study, we developed a new method of grid partitioning image analysis (GPIA) that quantifies the compositional heterogeneity of bacterial aggregates. Using Tol 5 derivatives that display AtaA and distinct fluorescent reporters, we demonstrate that GPIA (i) distinguishes between aggregated and dispersed cells, (ii) evaluates the heterogeneity of cell aggregates, and (iii) detects the changes in interaction affinity caused by modulating the production level of AtaA and the in-frame deletion of AtaA.

## 2 Materials and methods

### 2.1 Bacterial strains and culture conditions

*Escherichia coli* XL10-Gold and DH5α were used for plasmid construction. *E. coli* S-17 strain was used for plasmid conjugation (Simon et al., 1983). *E. coli* cells were grown in lysogeny broth (LB) medium at 37°C with shaking at 150 rpm. *Acinetobacter* sp. Tol 5 and its Δ*ataA* mutant strain 4,140 (Tol 5 Δ*ataA*) (Yoshimoto et al., 2023) were grown in LB medium at 28°C with shaking at 115 rpm. Antibiotics were added at the following concentrations as needed: ampicillin (100 μg/mL), kanamycin (50 μg/mL), gentamicin (10 μg/mL). L-arabinose was added to the culture medium to induce the expression of *ataA* gene on pAXG or pAXR plasmids. The concentration of arabinose varied from 0.01% to 0.5% (w/v) to control the production levels of AtaA.

### 2.2 DNA manipulation

Plasmids used in this study are listed in Table 1. The DNA fragment encoding the enhanced green fluorescent protein (*egfp*) gene was amplified by PCR from pEGFP-C3 (GenBank: U57607.1) using primers EGFPtoC007-Fw/EGFPtoC007-Rv (Fw: CAATTAAGCTTATGGTGAGCAAGGGCGAGG, Rv: GTATTCCATGGTTACTTGTACAGCTCGTCCATGC). The plasmid backbone including the P_LtetO1_ promoter for constitutive gene expression was amplified by PCR from pC007 (Abudayyeh et al., 2016) using primers C007inv-Fw/C007inv-Rv (Fw: GTATTCCATGGTAAGGATCTCCAGGCATCAAATAAAAC, Rv: CATTAAAGCTTTTTCTCCTCTTTCAGATCCGTGC). These DNA fragments were digested with HindIII and NcoI and ligated, generating pC007G. To construct pAXG and pAXR, which encode *egfp* or *mrfp* under the P_LtetO1_ promoter (Lutz and Bujard, 1997), the DNA fragments encoding *egfp* and *mrfp* were amplified by PCR from pC007G or pC007 using primers RFPtoARP-Fw/RFPtoARP-Rv (Fw: CAATTAAGCTTATGGTGAGCAAGGGCGAGG, Rv: GTATTCCATGG TTACTTGTACAGCTCGTCCATGC). The amplified DNA fragments were assembled with PvuII-digested pARP3 by NEBuilder HiFi DNA Assembly master mix (New England BioLabs, Ipswich, MA, United States). To construct pAXG-*ataA* and pAXR-*ataA*, which were used for co-expression of *ataA* and each fluorescent protein gene, the DNA fragments encoding *ataA* genes excised from pAtaA by digestion with EcoRI and XbaI were ligated with pAXG or pAXR digested with the same restriction enzymes. The construction of the co-expression plasmids for the in-frame deletion mutant of *ataA* and fluorescent protein was conducted in the same manner using pARP3 plasmid harboring a gene encoding each in-frame deletion mutant of *ataA* (Yoshimoto et al., 2023). Transformation of the Tol 5Δ*ataA* with these expression plasmids was carried out by conjugal transfer from *E. coli* S17-1 strain (Simon et al., 1983), as previously described (Ishikawa et al., 2012).

**Table 1:** Plasmids used in this study.

Plasmids	Description	Reference
pC007	Vector encoding *mrfp* under P_LtetO1_ promoter, Amp^R^	Abudayyeh et al. (2016)
pC007G	Vector encoding *egfp* under P_LtetO1_ promoter, Amp^R^	This study
pEGFP-C3	Vector encoding *egfp*, Kan^R^	Purchased from Takara Bio (Shiga, Japan)
pARP3	Expression vector for *E. coli* and *Acinetobacter*, Gm^R^, Amp^R^	Ishikawa et al. (2012)
pAtaA	Expression vector encoding *ataA* under AraC/P_BAD_ promoter, Amp^R^, Gm^R^	Ishikawa et al. (2012)
pAXG	Expression vector encoding *egfp* under P_LtetO1_ promoter, Amp^R^, Gm^R^	This study
pAXR	Expression vector encoding *mrfp* under P_LtetO1_ promoter, Amp^R^, Gm^R^	This study
pAXG-AtaA	Expression vector encoding *egfp* under P_LtetO1_ promoter and *ataA* under AraC/P_BAD_ promoter, Amp^R^, Gm^R^	This study
pAXR-AtaA	Expression vector encoding *mrfp* under P_LtetO1_ promoter and *ataA* under AraC/P_BAD_ promoter, Amp^R^, Gm^R^	This study
pAXG-ΔNhead	pAXG vector encoding *ataA* fragment carrying an in-frame deletion of 60–313 aa	This study
pAXG-ΔNS-A1	pAXG vector encoding *ataA* fragment carrying an in-frame deletion of 327–506 aa	This study
pAXG-ΔNS-A2	pAXG vector encoding *ataA* fragment carrying an in-frame deletion of 507–1,337 aa	This study
pAXG-ΔNS-B	pAXG vector encoding *ataA* fragment carrying an in-frame deletion of 1,338–2,335 aa	This study
pAXG-ΔNS-C-ΔChead	pAXG vector encoding *ataA* fragment carrying an in-frame deletion of 2,397–3,169 aa	This study
pAXG-ΔCstalk	pAXG vector encoding *ataA* fragment carrying an in-frame deletion of 3,170–3,475 aa	This study

### 2.3 Detection of co-expression of AtaA and fluorescent protein

Protein production was examined by SDS-PAGE followed by Coomassie Brilliant Blue (CBB) staining and immunoblotting using anti-AtaA_59-325_ antiserum, as described previously (Ishikawa et al., 2012).

The presentation of AtaA on the cell surface and the production of fluorescent proteins were confirmed by immunofluorescence microscopy using anti-AtaA_59-325_ antiserum, as described previously with a slight modification (Yoshimoto et al., 2023). Alexa Fluor 647 conjugate of anti-rabbit IgG (H + L), F(ab’)_2_ Fragment (Cell Signaling Technology, Danvers, MA, United States) was used for the detection of the primary antiserum. These prepared samples were observed by a confocal laser scanning microscope (CLSM; FV1000D IX81-FD/NIH, Olympus Corporation, Tokyo, Japan) with 473, 559, and 635 nm lasers. Confocal images were acquired using a 100×/1.4 NA oil immersion objective lens and saved at a resolution of 1,024 × 1,024 pixels.

Tube-settling aggregation assays of bacterial cells were performed as described previously (Ishikawa et al., 2012). In brief, glass test tubes containing cell suspension with an optical density at 660 nm (OD_660_) of 0.5 were left to stand at 28°C. The aggregation ratio was calculated from the decrease in the OD_660_ of the cell suspension using the following equation:

Aggregation ratio (%) = 100 × (Initial OD_660_ – OD_660_ after standing) / Initial OD_660_.

### 2.4 Formation of cell aggregation

Grown cells were transferred to a 15-mL protein low adsorption tube (Proteosave SS; Sumitomo Bakelite, Tokyo, Japan) and diluted to OD_660_ = 0.5 with fresh medium. 0.5 mL of two cell suspensions containing different types of cells were mixed in a 1.5-mL protein low adsorption tube (Sumitomo Bakelite, Tokyo, Japan). For samples mixed at a 1:3 ratio, 0.25 mL and 0.75 mL of cell suspension were mixed. The mixed cell suspension was centrifuged at 5,000 × g for 5 min, and the supernatant was discarded. The cell pellet was rinsed with deionized water and centrifuged at 5,000 × g for 5 min. The cell pellet was re-suspended in equal volume of BS-N buffer (34.5 mM Na_2_HPO_4_, 14.7 mM KH_2_PO_4_, 15.5 mM K_2_SO_4_; pH 7.2). The cell suspension was slowly stirred at 8 rpm using a rotator (NRC-20D, Nissinrika, Tokyo, Japan) for 15 min to form cell aggregates. The suspension containing planktonic cells and cell aggregates was placed onto a glass slide and observed by CLSM. The lasers of 473 and 559 nm were used for exciting the fluorescent proteins EGFP and mRFP, respectively. Confocal images were acquired using a 100×/1.4 NA oil immersion objective lens and saved at a resolution of 1,024 × 1,024 pixels.

### 2.5 Grid partitioning image analysis

The CLSM images were imported into ImageJ (Schneider et al., 2012). EGFP and mRFP fluorescent signals were separated using the “Color Balance” function; individual fluorescent particles were detected with “Find Maxima,” and their coordinates were exported as a spreadsheet, where the field of view was subdivided into 2 μm-square grids. The pixel scale of the CLSM image was calibrated to micrometer units. Grids containing one or no cells were classified as dispersed, while those containing two or more cells were classified as aggregated. For every grid classified as aggregated, the proportion of EGFP-fluorescent cells (EGFP-ratio) was calculated by dividing the number of EGFP-fluorescent cells by the total number of fluorescent cells (EGFP + mRFP) within that region. The grids classified as aggregated were then categorized into five bins based on EGFP-ratio: 0 ≤ x < 20%, 20 ≤ x < 40%, 40 ≤ x < 60%, 60 ≤ x < 80%, and 80 ≤ x ≤ 100%. For each bin, the total number of EGFP and mRFP cells was summed across all grids classified as aggregated. These values were then normalized by the total number of fluorescent cells present in both grids classified as dispersed and aggregated, and the normalized percentages were plotted as histograms. Only the import of the data into ImageJ and the Excel template was done manually; all other calculations and classifications were automated. The template spreadsheet used for these calculations is available in the Supplementary File S1.

### 2.6 Control conditions for theoretical distributions

To interpret experimental histograms, theoretical EGFP-ratio distributions were generated for each of the control conditions under defined assumptions. For the dispersed control, every cell was assumed to occupy its own grid, yielding no grids assigned as aggregated and therefore a flat distribution of 0% across all EGFP-ratio bins. In the homo-aggregation control, we posited that each grid contained only a single cell type, while the overall field maintained a 1:1 mixture of EGFP- and mRFP-expressing cells; this produced a theoretical profile composed solely of the 0–20% and 80–100% bins, each contributing 50% of the total. For the hetero-aggregation control, the expected distributions were modeled with the binomial formula:


P(k;n,p)=C(n,k)×p^k×(1−p)^(n−k)

Where *n* is the number of cells in a grid, k is the number of EGFP-fluorescent cells, and *p* is the probability of selecting an EGFP-fluorescent cell. We set *p* = 0.5 for the equal-volume (1:1) mixture and *p* = 0.25 for the 1:3 mixture, calculated the EGFP-ratio (k/n × 100) for k = 0–n, and assigned each outcome to the standard five bins (0 ≤ x < 20%, 20 ≤ x < 40%, 40 ≤ x < 60%, 60 ≤ x < 80%, and 80 ≤ x ≤ 100%). This computation was repeated for *n* = 2–8, spanning the observed range of cells per grid. The resulting bin probabilities were combined with the weights based on the observed frequency of each grid size, generating the theoretical distributions.

### 2.7 Statistical analysis

Statistical differences in the distribution of EGFP-ratio categories among groups were evaluated using Pearson’s chi-square test. For each pairwise comparison between experimental groups, contingency tables were constructed using the number of fluorescent cells in each bin (0–20%, 20–40%, 40–60%, 60–80%, 80–100%, and Dispersed). Expected frequencies were calculated under the null hypothesis of independence.

The chi-square statistics were calculated using the formula:


$$\chi^{2}=\sum (\frac{{\left(O-E\right)}^{2}}{E})$$

where O is the observed count and E is the expected count for each cell.

The degrees of freedom (df) were calculated as:


df=(r−1)×(c−1)

Where r is the number of groups being compared (rows), and c is the number of categories (columns).

The strength of association was assessed using Cramér’s V, defined as:


$$V=\sqrt{\frac{\chi^{2}}{N(k-1)}}$$

Where N is the total number of observations and k is the smaller of the number of rows or columns in the contingency table.

To correct for multiple comparisons, Bonferroni-adjusted *p*-values were calculated by multiplying the raw *p*-values by the number of comparisons.

## 3 Results

### 3.1 Construction of bacterial cells co-expressing *ataA* and two types of fluorescent protein genes

**Figure 1:**
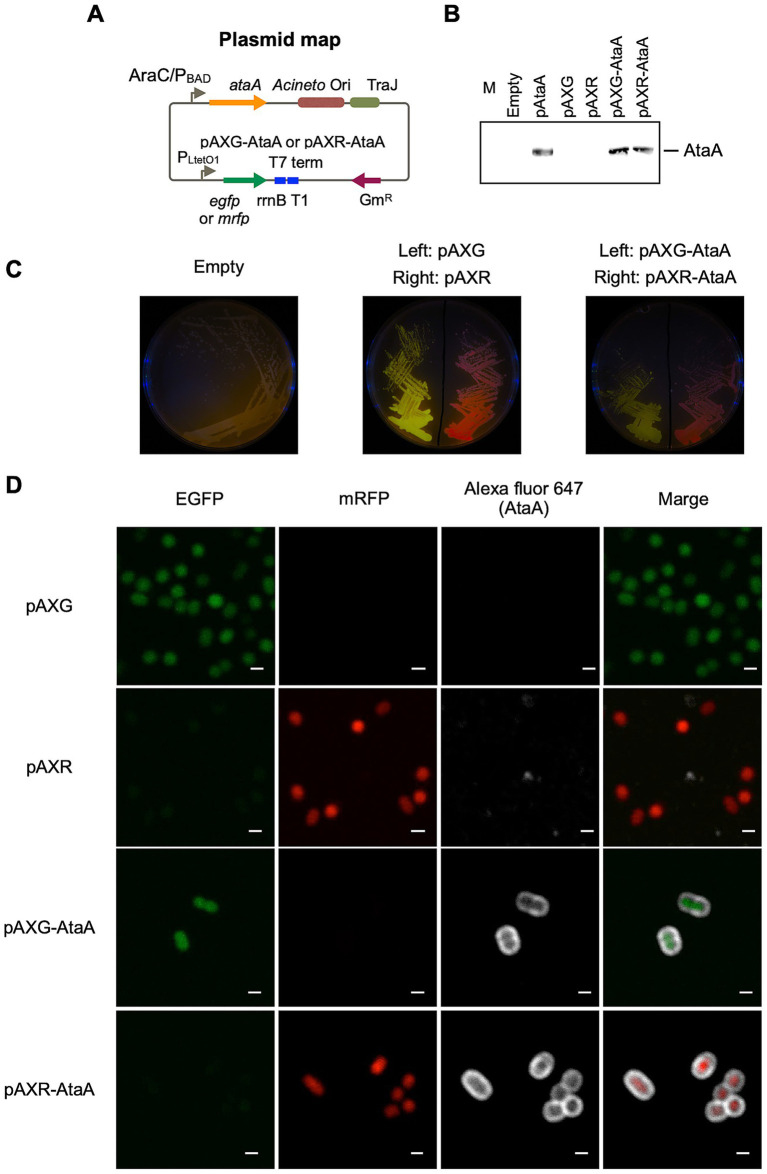
Construction of AtaA/EGFP or AtaA/mRFP co-expression strain. **(A)** Map of the co-expression plasmid for AtaA and fluorescent protein genes. The *ataA* gene was inserted under the AraC-P_BAD_ inducible promoter, and *egfp* or *mrfp* were inserted under the P_LtetO1_ constitutive promoter. **(B)** Confirmation of *ataA* expression. The whole-cell lysates of Tol 5 Δ*ataA* or its derivative mutants were analyzed by immunoblotting using anti-AtaA antiserum. **(C)** Photographs of agar plates taken under black light. Green and red are fluorescence derived from EGFP and mRFP, respectively. **(D)** CLSM observation of immuno-stained cells co-expressing *ataA* and fluorescent protein genes. Green and red are derived from EGFP and mRFP, respectively. White was derived from immuno-stained AtaA. Confocal images were acquired using a 100×/1.4 NA oil immersion objective lens with a 3 × digital zoom and saved at a resolution of 1,024 × 1,024 pixels. Scale bars: 1 μm.

First, we constructed *ataA* and fluorescent gene co-expressing Tol 5 cells to distinguish the two types of cells in the image analysis. The co-expression plasmids were designed and constructed as shown in Figure 1A and Table 1. Either *egfp* and *mrfp* genes were placed under the constitutive P_LtetO1_ promoter, while the *ataA* gene was placed under the inducible AraC/P_BAD_ promoter.

The constructed plasmids were introduced into Tol 5 Δ*ataA*, and the co-expression of each fluorescent protein gene and *ataA* was examined by immunoblotting and immunofluorescence microscopy. The production amount of AtaA and cell adhesiveness of Tol 5 Δ*ataA* (pAXG-AtaA) (EGFP-AtaA(+)) and Tol 5 Δ*ataA* (pAXR-AtaA) (mRFP-AtaA(+)) were almost the same as Tol 5 Δ*ataA* (pAtaA) (Figure 1B). Tol 5 Δ*ataA* (pAXG) (EGFP-AtaA(−)) and Tol 5 Δ*ataA* (pAXR) (mRFP-AtaA(−)) exhibited intracellular fluorescence corresponding to each fluorescent protein (Figures 1C,D). Tol 5 Δ*ataA* (pAXG-AtaA) and Tol 5 Δ*ataA* (pAXR-AtaA) simultaneously exhibited fluorescence surrounding the cells corresponding to AtaA in addition to intracellular fluorescence corresponding to each fluorescent protein (Figure 1D). These results demonstrated that each fluorescent protein gene and *ataA* were co-expressed in Tol 5 Δ*ataA*.

### 3.2 Evaluation of cell aggregation and heterogeneity by grid partitioning image analysis (GPIA)

**Figure 2:**
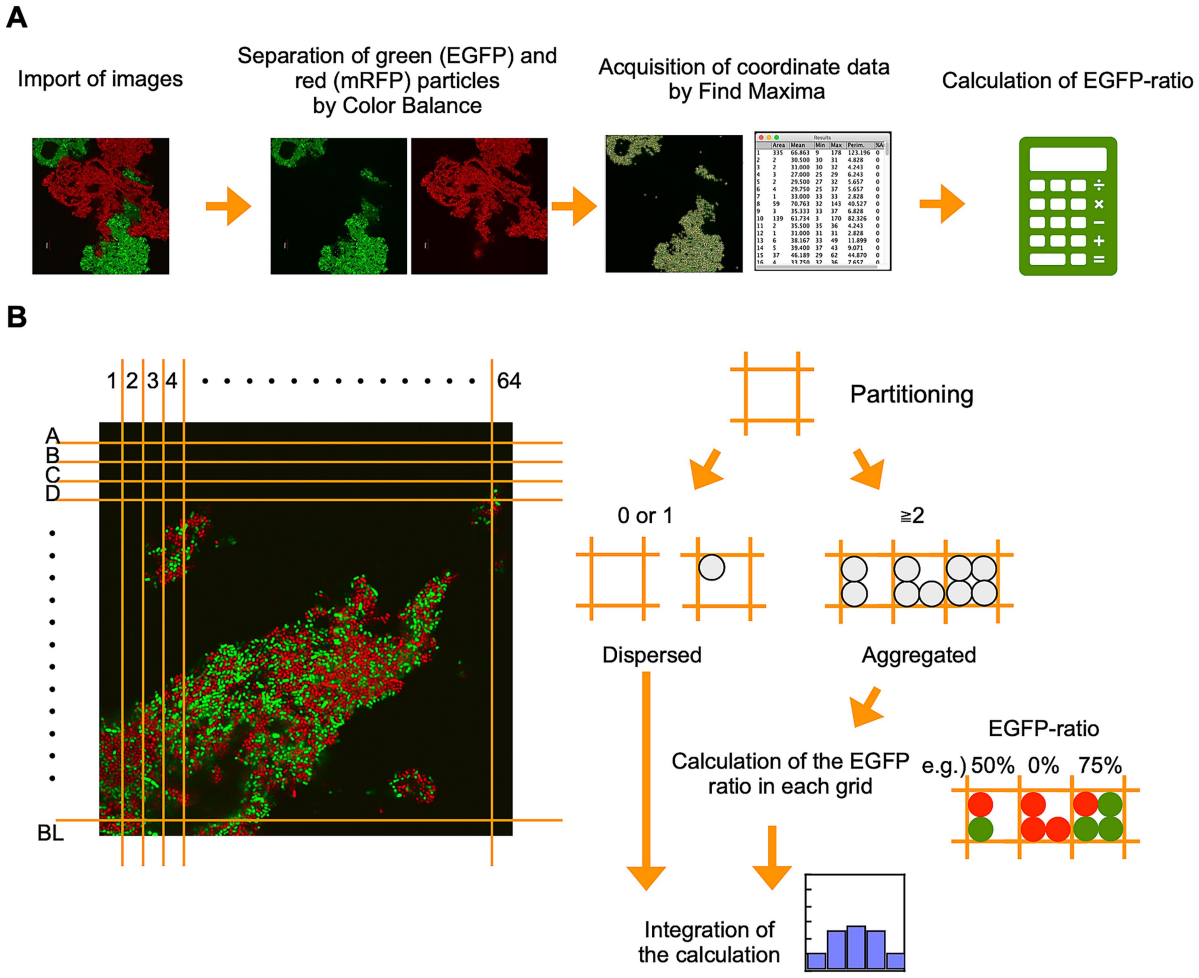
Overview of grid partitioning image analysis (GPIA). **(A)** CLSM images were imported into ImageJ and separated into EGFP and mRFP channels using the “color balance” function. Fluorescent cell coordinates were extracted independently from each channel using the “Find Maxima” function, and the resulting coordinate sets were exported as a spreadsheet for further analysis. **(B)** Concept of grid partitioning and cell classification. Based on the imported cell coordinates, the image was divided into 64 × 64 grids (2 μm × 2 μm). A grid containing one or no cells was classified as “dispersed,” while grids containing multiple cells were defined as “aggregated.” For each aggregated grid, the proportion of EGFP-fluorescent cells was calculated and binned into one of five EGFP-ratio ranges: 0 ≤ x < 20%, 20 ≤ x < 40%, 40 ≤ x < 60%, 60 ≤ x < 80%, and 80 ≤ x ≤ 100%. The number of EGFP and mRFP cells within each bin was summed across all aggregated grids. The total number of cells across both dispersed and aggregated grids was used as the normalization baseline (100%), and the binned values were plotted as percentage histograms.

For the previous analysis of eukaryotic cells by Sieber and Roseman, each four-cell aggregate was treated as a single analytical unit (Sieber and Roseman, 1981). Here, we investigated the optimal subdivision size for CLSM images to divide aggregates of the coccobacillus-shaped bacterial cells, whose diameter is approximately 0.8–1.2 μm (Figure 1D), into units of about four cells. We divided the CLSM image of well-mixed hetero-aggregated cells shown in Figure 2B into square grids of 1, 2, and 4 μm and counted the number of cells in each grid. In 1 μm square grids, over 70% of the cells were present individually within a single grid (Supplementary Figure S1, green). Because we defined “aggregated” as a grid containing two or more cells, 1 μm partitioning led to misclassification. In 4 μm square grids, many grids contained ten or more cells (Supplementary Figure S1, blue), so cells that were not in direct contact could be falsely classified as co-aggregated. In 2 μm square grids, most of the grids contained two to four cells (Supplementary Figure S1, orange), which were correctly classified as aggregated. These results indicate that the 2 μm partitioning is optimal for this bacterial cell, which is reasonable because a 2 μm square grid accommodates roughly four 1 μm-diameter cells. Therefore, all subsequent analyses used a 2 μm grid.

**Figure 3:**
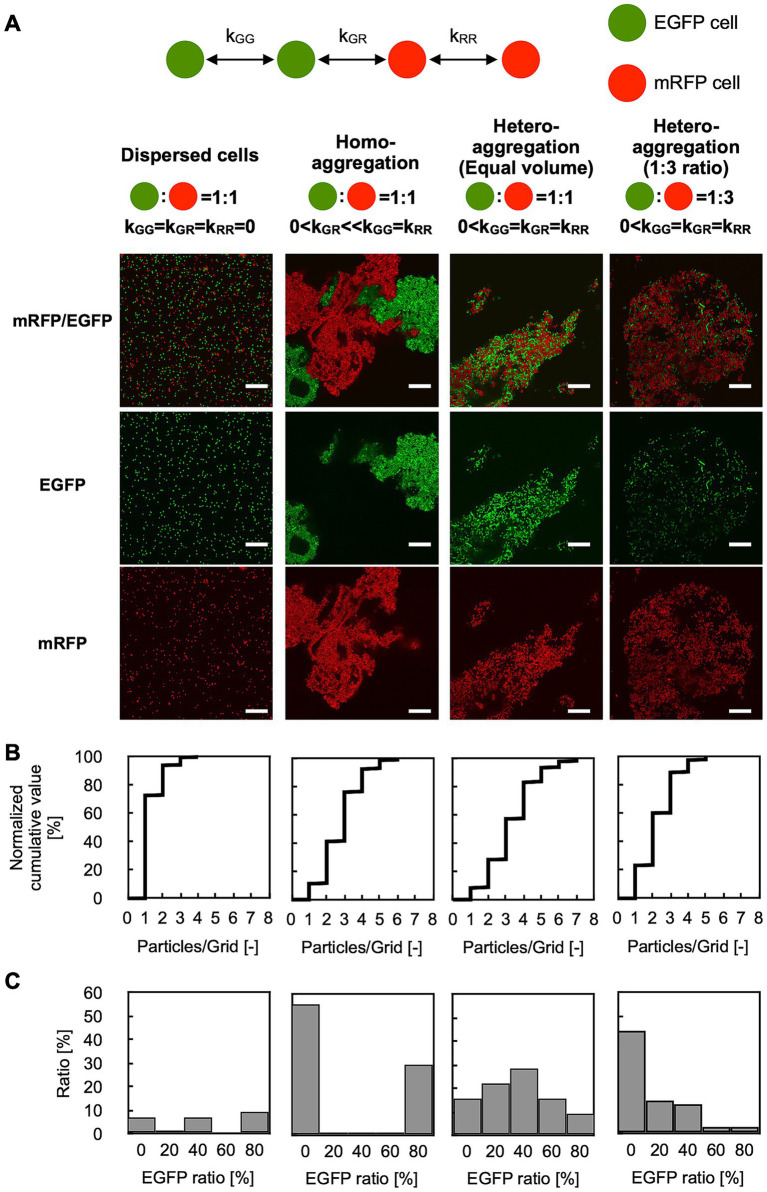
GPIA of dispersed or aggregated cells. **(A)** CLSM images and schematic cartoons of four control samples: dispersed cells composed of EGFP-AtaA(−) + mRFP-AtaA(−); homo-aggregation obtained by first forming separate EGFP-AtaA(+) and mRFP-AtaA(+) clumps and then mixing them; and hetero-aggregation generated by mixing equal (1:1) or unequal (1:3) volumes of EGFP-AtaA(+) and mRFP-AtaA(+) cells. Confocal images were acquired using a 100×/1.4 NA oil immersion objective lens and saved at a resolution of 1,024 × 1,024 pixels. Scale bars: 20 μm. **(B)** Frequency distribution of total cell counts in each 2 μm square grid. **(C)** Ratio of the EGFP-fluorescent cells in each grid containing multiple cells (≥ 1,000 cells analyzed per sample) calculated from a single field of view. Data shown are representative of at least two independent biological experiments that yielded similar results.

To test the performance of GPIA, four typical images were prepared by mixing the two types of AtaA-displayed (or not displayed) fluorescent cells. The cell mixture of EGFP-AtaA(−) and mRFP-AtaA(−) was prepared as the dispersed control (Figure 3A left). The second control consisted of two cell types that can self-aggregate individually but do not interact with each other; we referred to this as the homo-aggregation control. This sample was prepared by forming homotypic cell aggregates of EGFP-AtaA(+) and mRFP-AtaA(+) cells and then mixing them (Figure 3A middle left). The third and fourth controls consisted of two cell types that interacted with one another and with themselves to a similar extent; we designated this as the hetero-aggregate control (Figure 3A middle right and right). These samples were prepared by mixing equal or 1:3 volumes of EGFP-AtaA(+) and mRFP-AtaA(+) cell suspensions, allowing simultaneous aggregation. These samples were observed by CLSM and analyzed according to the GPIA concept presented in Figure 2. In the dispersed control, over 70% of cells were classified as dispersed particles (Figure 3B, left). On the other hand, over 75% of cells were classified as aggregates in the homo- and hetero-aggregation controls (Figure 3B, middle left, middle right, and right).

Next, the ratio of EGFP-fluorescent cells in each grid was calculated (Figure 3C). In this calculation, particles were integrated as over 1,000 events from several image samples because the deviation was almost saturated at over 300 events (Supplementary Figure S2). In the dispersed control, no peak was observed because most particles were classified as a single cell in Figure 3B. On the other hand, the homo-aggregation control showed a U-shaped histogram, with high frequencies in the 0–20% and 80–100%, indicating that multiple cells of one type are included in each grid. In contrast, the hetero-aggregation control with a 1:1 ratio showed a symmetric unimodal histogram with the peak at 40–60%, indicating that two types of multiple cells equally contained in each grid. The hetero-aggregation control with a 1:3 ratio showed a right-skewed histogram, indicating that grids containing one cell type and grids containing two cell types were both present. All pairwise comparisons among the four samples (dispersed cells, homo-aggregates, hetero-aggregates with equal volume, and hetero-aggregates at a 1:3 ratio) showed statistically significant differences in the distribution of the EGFP-ratio, as determined by Pearson’s chi-square test with Bonferroni correction (Supplementary Table S1). Supplementary Figure S3 presents the theoretically calculated EGFP-ratio histogram, which displays a distribution closely matching the trend observed in the GPIA-generated histogram. These results demonstrate that GPIA not only distinguishes dispersed from aggregated cell populations but also clearly resolves whether the aggregates arise from homotypic or heterotypic interactions.

### 3.3 Analysis of cell–cell interaction mediated by cells expressing different levels of AtaA

**Figure 4:**
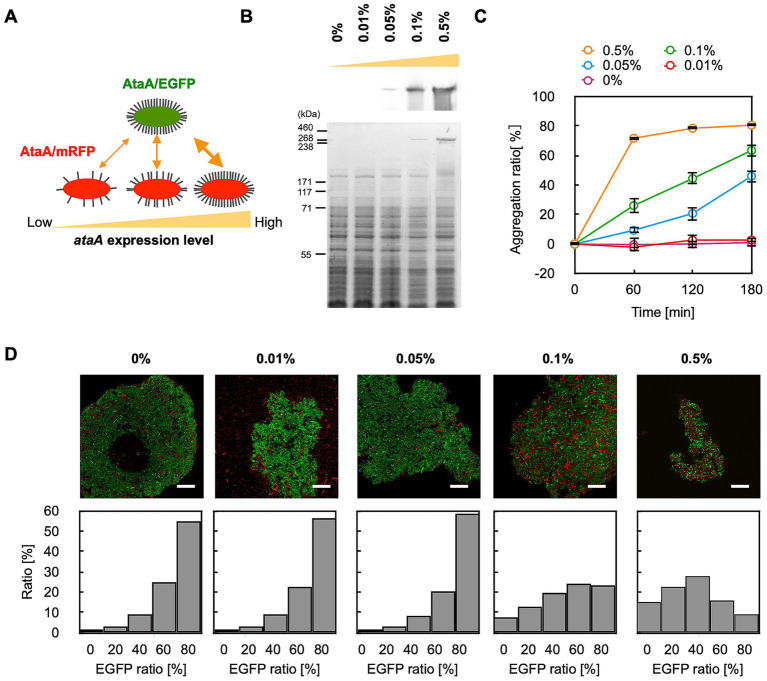
GPIA of co-aggregation mediated by AtaA with different levels of expression. **(A)** Experimental design: EGFP-AtaA(+) cells induced with 0.5% arabinose were mixed with mRFP-AtaA(+) cells induced with 0, 0.01, 0.05, 0.1, or 0.5% arabinose. **(B)** Immunoblotting and CBB staining of stepwise increases in AtaA production with higher arabinose concentrations. **(C)** Tube-settling assay of cells induced with 0, 0.01, 0.05, 0.1, or 0.5% arabinose. The data are presented as the means ± SDs (*n* = 3). **(D)** Representative merged CLSM images and corresponding EGFP-ratio histograms calculated from five fields of view by GPIA (≥ 1,000 cells per sample). Confocal images were acquired using a 100×/1.4 NA oil immersion objective lens and saved at a resolution of 1,024 × 1,024 pixels. Data shown are representative of at least two independent biological experiments that yielded similar results. Scale bars: 20 μm.

Next, we examined whether the GPIA could detect the difference in the interaction strength between bacterial cells. The mRFP-AtaA(+) cells were cultured in the medium containing 0–0.5% arabinose to prepare AtaA-displayed cells with different expression levels (Figure 4A). Immunoblotting showed that the expression level of *ataA* increased according to the arabinose concentration of over 0.05% (Figure 4B). Consistent with this, tube-settling assays showed that cells in which AtaA was detectable exhibited self-aggregation, and the aggregation rate increased with higher AtaA levels (Figure 4C).

We then mixed EGFP-AtaA(+) cells grown in 0.5% arabinose with each mRFP-AtaA(+) grown in 0–0.5% arabinose and prepared cell aggregates. GPIA revealed that the proportion of hetero-aggregated grids decreased progressively as the AtaA content of the mRFP cells declined (Figure 4D; Supplementary Table S2). Notably, cells induced with only 0.05% arabinose, which expressed little AtaA and aggregated slowly, were scarcely incorporated into the rapidly forming EGFP-AtaA cell aggregates. These results demonstrate that GPIA can detect expression-dependent differences in cell–cell interaction affinity.

### 3.4 Analysis of cell–cell interaction mediated by in-frame deletion mutants of AtaA

**Figure 5:**
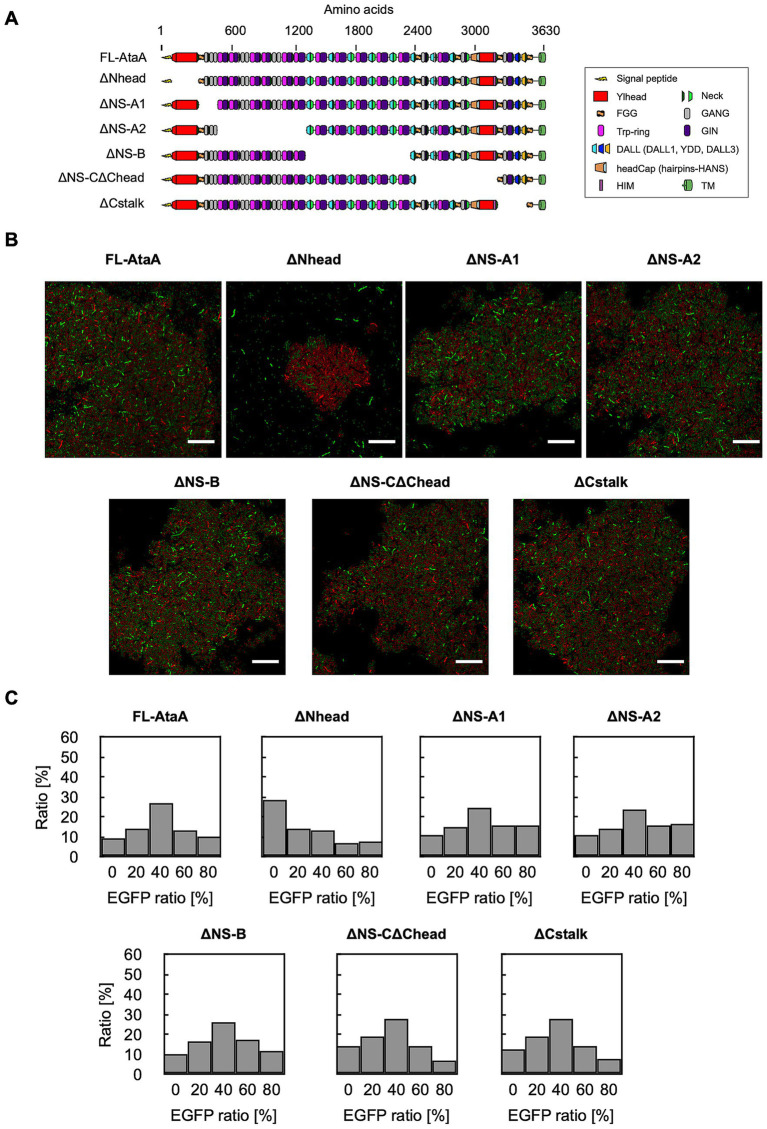
GPIA of cell–cell interaction mediated by in-frame deletion mutants of AtaA. **(A)** Schematic illustration of full-length AtaA (FL-AtaA) and the in-frame deletion mutants. **(B)** CLSM images of mixtures containing cells expressing EGFP and AtaA-mutants and cells expressing mRFP and FL-AtaA. Confocal images were acquired using a 100×/1.4 NA oil immersion objective lens and saved at a resolution of 1,024 × 1,024 pixels. Scale bars: 20 μm. **(C)** EGFP-ratio histograms calculated from five fields of view by GPIA (≥ 1,000 cells per sample). A chi-square test confirmed a significant difference between the ΔNhead and the other mutants (see Supplementary Table S3). Data shown are representative of at least two independent biological experiments that yielded similar results.

Previously, we showed that removing the N-terminal head domain (Nhead) significantly decreased cell adhesion to material surfaces, whereas deleting other parts did not. However, the domain responsible for self-aggregation was still unknown. To identify it, we built cells expressing EGFP and in-frame deletion mutants of AtaA that lack each domain (Figure 5A) and mixed them with cells expressing mRFP and full-length AtaA (FL-AtaA). We then observed aggregates with CLSM and analyzed them by GPIA (Figures 5B,C). The cells expressing ΔNS-A1, ΔNS-A2, ΔNS-B, ΔNS-CΔChead, and ΔCstalk formed mixed clumps with cells expressing FL-AtaA and the EGFP-ratio histograms showed a peak at 40–60%, indicating that aggregates comprised the two types of cells in a 1:1 ratio. In contrast, the ΔNhead mutant failed to co-aggregate—only the cells expressing FL-AtaA aggregated, while the ΔNhead cells stayed dispersed. The EGFP-ratio histogram of ΔNhead showed a peak at 0–20%, indicating that aggregates were made almost exclusively of mRFP-AtaA(+) cells. A chi-square test showed a significant difference between ΔNhead and the others in the distribution of EGFP-ratio (Supplementary Figure S3). These results suggest that cell–cell interaction mediated by AtaA is driven mainly by homophilic interactions between Nhead of two different cells.

## 4 Discussion

In this study, we developed grid partitioning image analysis (GPIA) that transforms confocal micrographs into quantitative data and resolves both the presence of bacterial aggregates and the composition of the aggregates. The method correctly separated four reference conditions: fully dispersed suspensions, homo aggregates, and two hetero aggregate mixtures (Figure 1). Because GPIA relies only on standard ImageJ functions and a spreadsheet template, the workflow can be completed in minutes without custom code, making it an easy and accessible method. Compared with classical tube-settling assays that monitor turbidity, GPIA overcomes a key limitation: it can measure interactions among strains that are self-aggregative. GPIA enabled detailed analysis of the changes in cell aggregation resulting from variations in AtaA expression levels and from mutations (Figures 4, 5). These results suggest that GPIA is useful for analyzing adhesin mutants, environmental cues, or inhibitory compounds that produce modest phenotypic changes.

In Figure 4, we showed that GPIA detects changes in cell–cell affinity when AtaA expression is modulated with arabinose. A previous proteomic study of Tol 5 ranked AtaA as the second most abundant protein among the 1,977 proteins detected (Inoue et al., 2025). Other TAAs that promote aggregation have likewise been observed in large copy numbers on the cell surface (Hoiczyk et al., 2000; Kaiser et al., 2012), suggesting that TAA-mediated aggregation depends on a high surface density of the adhesin and likely involves cooperative interactions among multiple molecules. Our further analysis using the in-frame deletion mutants suggests that interactions between Nhead domains cause the cell aggregation (Figure 5). Comparable N-terminal head-mediated, zipper-like interactions have been reported for some well-characterized TAAs, *Yersinia enterocolitica* YadA and *Bartonella henselae* BadA (Hoiczyk et al., 2000; Kaiser et al., 2012). Because Nhead of AtaA also mediates adhesion to abiotic surfaces (Yoshimoto et al., 2023), this domain can be regarded as a multifunctional domain that mediates both cell–cell aggregation and initial surface adhesion.

By adjusting the grid size, GPIA can be readily applied to aggregation analyses of bacteria with different sizes. For instance, a 4-μm grid is likely appropriate for cells with a diameter of about 2 μm (e.g., some species of *Deinococcus*, *Sarcina*, and *Aquisphaera*) (Bondoso et al., 2011; Floc'h et al., 2019; Marcelino et al., 2021). When examining mixed populations of markedly different sizes, such as bacteria and yeast, it may be necessary to re-optimize both the grid size and the classification thresholds. Cell shape and orientation can also influence the analysis. In this study, local fluorescence intensity peaks were detected with the ImageJ “Find Maxima” function and converted to coordinates. As a result, cell orientation was not considered, and each peak was automatically regarded as the cell centroid. Under our culture conditions (stationary phase), Tol 5 exhibited a nearly spherical coccobacillary morphology (Figure 2D). Consequently, in the observation fields of aggregated cells, as shown in Figure 1B and Supplementary Figure S1, roughly 2 to 8 cells fell within each 2-μm square grid, enabling us to distinguish between hetero- and homo-aggregation. Thus, for a coccobacillus such as Tol 5, cell orientation is unlikely to affect the analysis substantially. In more elongated cells, however, the gap between the actual area occupied by the cell and the centroid derived from the fluorescence peak is expected to widen, lowering analytical accuracy. Therefore, GPIA is considered suitable for cocci and short, nearly spherical rods (coccobacilli).

In GPIA, a small proportion of physically proximate but non-interacting cells may sometimes be counted as aggregates. This can be minimized by diluting the cell suspension before CLSM observation and analyzing a sufficiently large number of fields. When increasing sample size, it is better to increase the number of observed fields rather than increase cell density because high-concentration samples cause misclassification simply due to spatial crowding. Further statistical analysis of the histogram of EGFP-ratio between samples using Pearson’s chi-square test would help to consider the significant differences.

In conclusion, we developed the GPIA, a rapid, simple, and sensitive method for quantitative analysis of bacterial cell aggregation. By converting confocal images into robust numerical data, GPIA bridges the gap between qualitative microscopy and quantitative, yet technically demanding, single-cell analysis. GPIA will accelerate research on cell–cell interactions, which are the basis of important bacterial functions such as surface colonization, tolerance to environmental stress, and interspecies metabolite exchange.

## Data Availability

The original contributions presented in the study are included in the article/Supplementary material, further inquiries can be directed to the corresponding author.
