# Supplementary material for: Grid partitioning image analysis of highly aggregative bacterium Acinetobacter sp. Tol 5
Source: Front Microbiol. 2025 Sep 10;16:1637462. doi: 10.3389/fmicb.2025.1637462 (PMC12459662; doi:10.3389/fmicb.2025.1637462)
Supplement: Supplementary file 1 [file Data_Sheet_1.PDF]

Supplementary Material for

Grid partitioning image analysis for bacterial cell aggregates

Yuki Ohara[1†], Shogo Yoshimoto[2†], Katsutoshi Hori[2,*]

[1] Friend Microbe Inc., Nagoya, Japan.

[2] Department of Biomolecular Engineering, Graduate School of Engineering, Nagoya University, Nagoya, Japan

[†] These authors contributed equally to this work and share first authorship.

*Correspondence: khori@chembio.nagoya-u.ac.jp

Phone: +81-52-789-3339

Fax: +81-52-789-3218

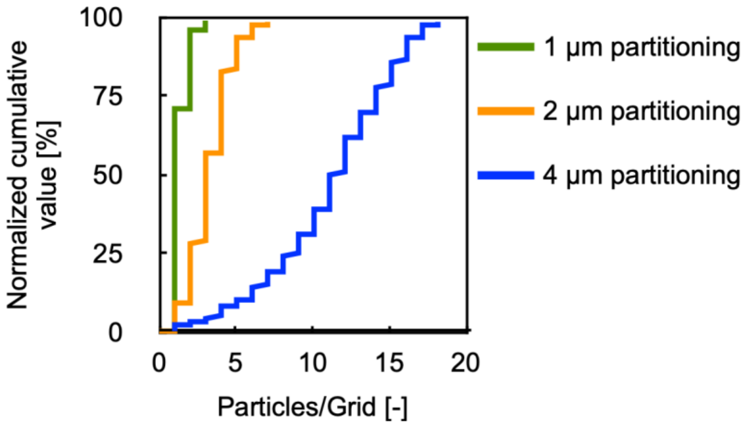

Figure S1. Particle numbers in each grid.

The particle counts in each grid was integrated when the partitioning size was set to 1, 2, and 4 μm of a square. This analysis was performed on the hetero-aggregation control images shown in Figure 3A.

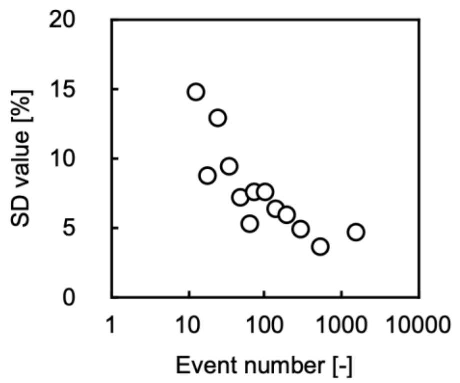

Figure S2. Calculation of the SD values of EGFP-ratio.

The horizon axis means the total number of analyzed particles. The vertical axis means the average SD values of each EGFP-ratio in histograms. This analysis was performed on the hetero-aggregation control images shown in Figure 3A.

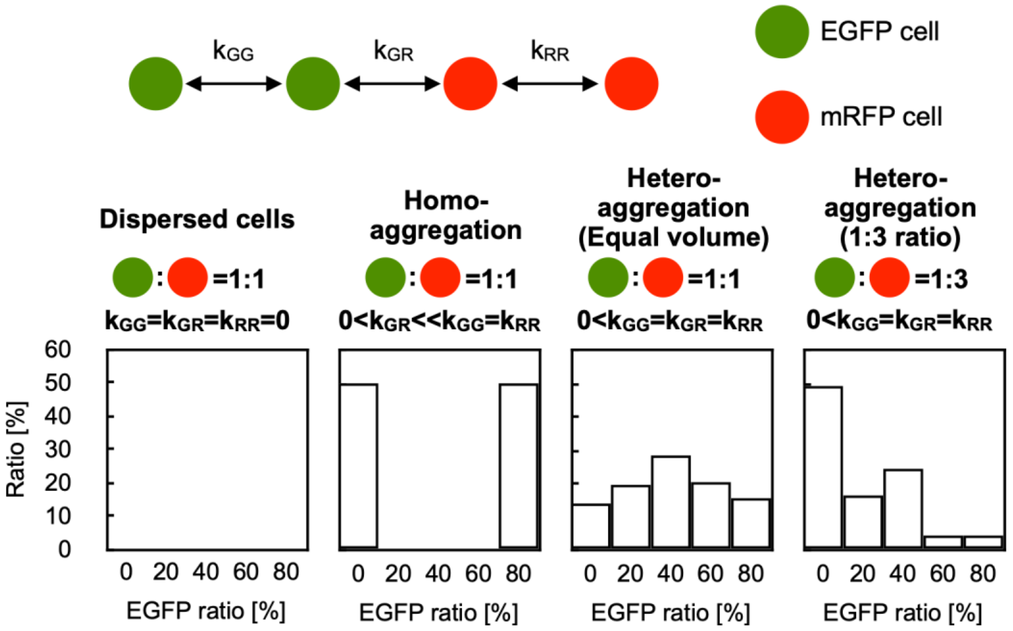

Figure S3. Theoretical values of EGFP-ratio.

The theoretical values of EGFP-ratio of each control sample in Figure 3 were calculated.

Table S1. Statistical comparisons of the EGFP-ratio in Figure 3C by Pearson's chi-square test with Bonferroni correction.

	Chi^2	p-value	Cramér's V	Bonferroni-adjusted p
Dispersed vs Homo	1884	$< 1.0e^{-300}$	0.697	$< 1.0e^{-300}$
Dispersed vs Hetero (1:1)	1606	$< 1.0e^{-300}$	0.597	$< 1.0e^{-300}$
Dispersed vs Hetero (1:3)	1230	$8.50e^{-264}$	0.518	$5.10e^{-263}$
Homo vs Hetero (1:1)	2522	$< 1.0e^{-300}$	0.659	$< 1.0e^{-300}$
Homo vs Hetero (1:3)	1816	$< 1.0e^{-300}$	0.555	$< 1.0e^{-300}$
Hetero (1:1) vs Hetero (1:3)	1121	$2.79e^{-240}$	0.415	$1.67e^{-239}$

Table S2. Statistical comparisons of the samples in Figure 4D by Pearson's chi-square test with Bonferroni correction.

	Chi^2	p-value	Cramér's V	Bonferroni-adjusted p
0.5-0.5% vs 0.5-0.1%	1045	$7.01e^{-224}$	0.175	$7.01e^{-223}$
0.5-0.5% vs 0.5-0.05%	5891	$< 1.0e^{-300}$	0.448	$< 1.0e^{-300}$
0.5-0.5% vs 0.5-0.01%	5086	$< 1.0e^{-300}$	0.495	$< 1.0e^{-300}$
0.5-0.5% vs 0.5-0%	7015	$< 1.0e^{-300}$	0.375	$< 1.0e^{-300}$
0.5-0.1% vs 0.5-0.05%	8069	$< 1.0e^{-300}$	0.376	$< 1.0e^{-300}$
0.5-0.1% vs 0.5-0.01%	5774	$< 1.0e^{-300}$	0.345	$< 1.0e^{-300}$
0.5-0.1% vs 0.5-0%	9756	$< 1.0e^{-300}$	0.354	$< 1.0e^{-300}$
0.5-0.05% vs 0.5-0.01%	50	$1.08e^{-09}$	0.032	$1.08e^{-08}$
0.5-0.05% vs 0.5-0%	188	$6.69e^{-39}$	0.050	$6.69e^{-38}$
0.5-0.01% vs 0.5-0%	39	$2.01e^{-07}$	0.023	$2.01e^{-06}$

Table S3. Statistical comparisons of the samples in Figure 5C by Pearson's chi-square test with Bonferroni correction.

	Chi^2	p-value	Cramér's V	Bonferroni-adjusted p
ΔNhead vs FL-AtaA	2976	$< 1.0e^{-300}$	0.251	$< 1.0e^{-300}$
ΔNhead vs ΔNS-A1	2722	$< 1.0e^{-300}$	0.287	$< 1.0e^{-300}$
ΔNhead vs ΔNS-A2	2857	$< 1.0e^{-300}$	0.282	$< 1.0e^{-300}$
ΔNhead vs ΔNS-B	2722	$< 1.0e^{-300}$	0.314	$< 1.0e^{-300}$
ΔNhead vs ΔNS-CΔChead	1974	$< 1.0e^{-300}$	0.252	$< 1.0e^{-300}$
ΔNhead vs ΔCstalk	2423	$< 1.0e^{-300}$	0.262	$< 1.0e^{-300}$

File S1. Template spreadsheet for GPIA.

Input the coordinates of EGFP and mRFP. The histogram of EGFP-ratio is output on the right side of the spreadsheet. A spreadsheet containing model data is also included.
